# Investigation on the cutting-infiltration integrated strategy based on medical waterjet for targeted drug delivery

**DOI:** 10.1038/s41598-026-39721-y

**Published:** 2026-02-19

**Authors:** Yukun Lan, Wenchuan Liu, Jiren Tang, Hong Li, Shuaikang Chang, Guiqiang Hao

**Affiliations:** 1 State Key Laboratory of Coal Mine Disaster Dynamics and Control, Chongqing University, Chongqing, 400044 China; 2 State and Local Joint Engineering Laboratory of Methane Drainage in Complex Coal Gas Seam, Chongqing University, Chongqing, 400044 China; 3 Department of Anesthesiology, The Second Affiliated Hospital of Army Medical University, Chongqing, 400044 China

**Keywords:** Medical water jet, Drug delivery, Anesthesia drug, Tissue damage, Nonmonotonic diffusion properties, Cancer, Engineering, Health care, Medical research, Oncology, Optics and photonics

## Abstract

The integration of cutting and anesthesia within a single surgical instrument presents a significant opportunity to increase precision and reduce mechanical injury. This study introduces an innovative integrated water jet system that synergistically combines tissue dissection with localized drug delivery. A dedicated experimental platform was developed on the basis of hydrodynamic principles, enabling synchronous cutting and anesthesia. Systematic evaluations were conducted through cutting-diffusion experiments, tissue surface morphology analysis, and spatial diffusion tracking of anesthetics via photoacoustic imaging. The results demonstrated a nonlinear positive correlation between jet parameters (pressure and nozzle diameter) and both the cutting depth and diffusion distance. The optimal performance was achieved at distinct parameters for different tissue types: 4 MPa with a 0.2 mm nozzle for muscle tissue, and 8 MPa with a 0.2 mm nozzle for adipose tissue. Compared with conventional scalpel excision, the water jet technique significantly reduced tissue damage, as evidenced by a 51% reduction in fiber breakage and a 35% decrease in damaged area, while preserving up to 39.45 μm of functional structure. Furthermore, photoacoustic imaging revealed nonmonotonic diffusion dynamics of the anesthetic, with the maximum diffusion distance occurring adjacent to the cutting depth (18.31 ± 2 mm), confirming a “cutting-guided diffusion” mechanism. These findings establish a foundational framework for device-drug synergy in surgery, advancing the development of multifunctional, minimally invasive technologies.

## Introduction

Soft tissue dissection is a fundamental procedure in surgery, yet balancing precise separation, hemostasis, and pain management remains a clinical challenge. Conventional instruments like scalpels lack hemostatic capability^1,2^, while electrosurgical devices often cause thermal collateral damage that delays healing^3^. Furthermore, intraoperative pain control typically relies on preoperative injection, which may not provide sustained analgesia during extensive dissections.

The medical application of water jets was first described by Papachristou and Barters^4,5^, who demonstrated their effectiveness in reducing intraoperative blood loss. Subsequent studies by Yoshie Une et al.^6,7^ and Baer et al.^8^ applied water jet resection to cirrhotic livers, hepatocellular carcinoma, and solid liver tumors, confirming that exposed intrahepatic elastic vessels could be protected from injury. Rau et al.^9^ further validated the clinical benefits of water jet dissection in open and laparoscopic liver surgeries, showing reduced blood loss, Pringle maneuver time, and resection duration compared with those of the CUSA and blunt dissection.

In recent years, advancements in fluid control technology have significantly expanded the application of waterjets across diverse surgical subspecialties. In urology, multi-center studies by Gilling et al.^10^ and Elterman et al.^11^ have validated the long-term safety and efficacy of robotic-assisted waterjet ablation (Aquablation) for benign prostatic hyperplasia, while Nguyen et al.^12^ further analyzed strategies for optimizing postoperative hemostasis. In the fields of endoscopy and general surgery, Cecinato et al.^13^ and Kotecha et al.^14^ confirmed the tissue selectivity and minimally invasive safety of waterjets in endoscopic submucosal dissection (ESD) and necrotizing pancreatitis debridement, respectively.

Furthermore, significant progress has been made in precision plastic and reconstructive surgery. Research by Wormald et al.^15^ and Shimada et al.^16^ indicated that hydrosurgery debridement maximizes the preservation of viable dermis in burn wounds. In the treatment of axillary osmidrosis, recent comparative studies by Xia et al.^17^ and Moon et al.^18^ highlighted the superiority of hydrosurgery over traditional excision in minimizing scarring and complications. In neurosurgery, where precision is paramount, Azab et al.^19^ demonstrated the ability of waterjet dissection to preserve microvascular structures during glioma resection. Concurrently, Zhao et al.^20^ and Babaiasl et al.^21^ established fluid-structure interaction models based on soft tissue fracture mechanics, enabling precise prediction of cutting depth. Meanwhile, in the parallel field of drug delivery, Srivastava et al.^22^ verified the efficiency of jet injectors for needle-free administration.

Despite these advances, current research prioritizes macroscopic clinical outcomes over histological mechanisms. Crucially, surgical systems continue to treat saline purely as a mechanical medium, ignoring the jet’s potential as a drug delivery vector. This limitation precludes the synergistic integration of dissection and anesthesia, necessitating inefficient separate steps.

To address this limitation, we propose an integrated cutting-anesthesia strategy based on the hydrodynamic principle of Cutting-Guided Diffusion. Theoretically, the mechanism operates on the duality of the jet stream: the high-velocity core possesses kinetic energy exceeding the tissue’s ultimate tensile strength to effectuate cutting, while the peripheral fluid, upon stagnation, generates a hydrostatic pressure gradient. This gradient actively drives the fluid—loaded with anesthetic agents—into the porous tissue matrix, potentially achieving immediate regional saturation synchronously with the incision.

Anesthetic efficacy is positively correlated with the extent of tissue diffusion^23,24^. Several studies have explored drug diffusion dynamics via various methodologies: Marcus Caine’s team implanted fluorescently labeled doxorubicin into gels, quantified its spatial distribution via fluorescence microscopy and confirmed a power-law decay in drug concentration^25^; Errol Mathias et al. investigated 5-fluorouracil diffusion in fluorinated PEG hydrogels via ¹H molecular diffusion NMR and ¹⁹F spin diffusion NMR, demonstrating that hydrophobic groups hinder drug migration^26^; Štefan Bálint’s team monitored drug diffusion via SERS probes after embedding silver nanoparticle-coated silica beads into cancer cells, successfully tracking the dynamic distribution of low-concentration drugs within organelles^27^. However, these methods are limited by shallow penetration depth, impeding comprehensive spatial studies of drug diffusion in tissues.

**Fig. 1:**
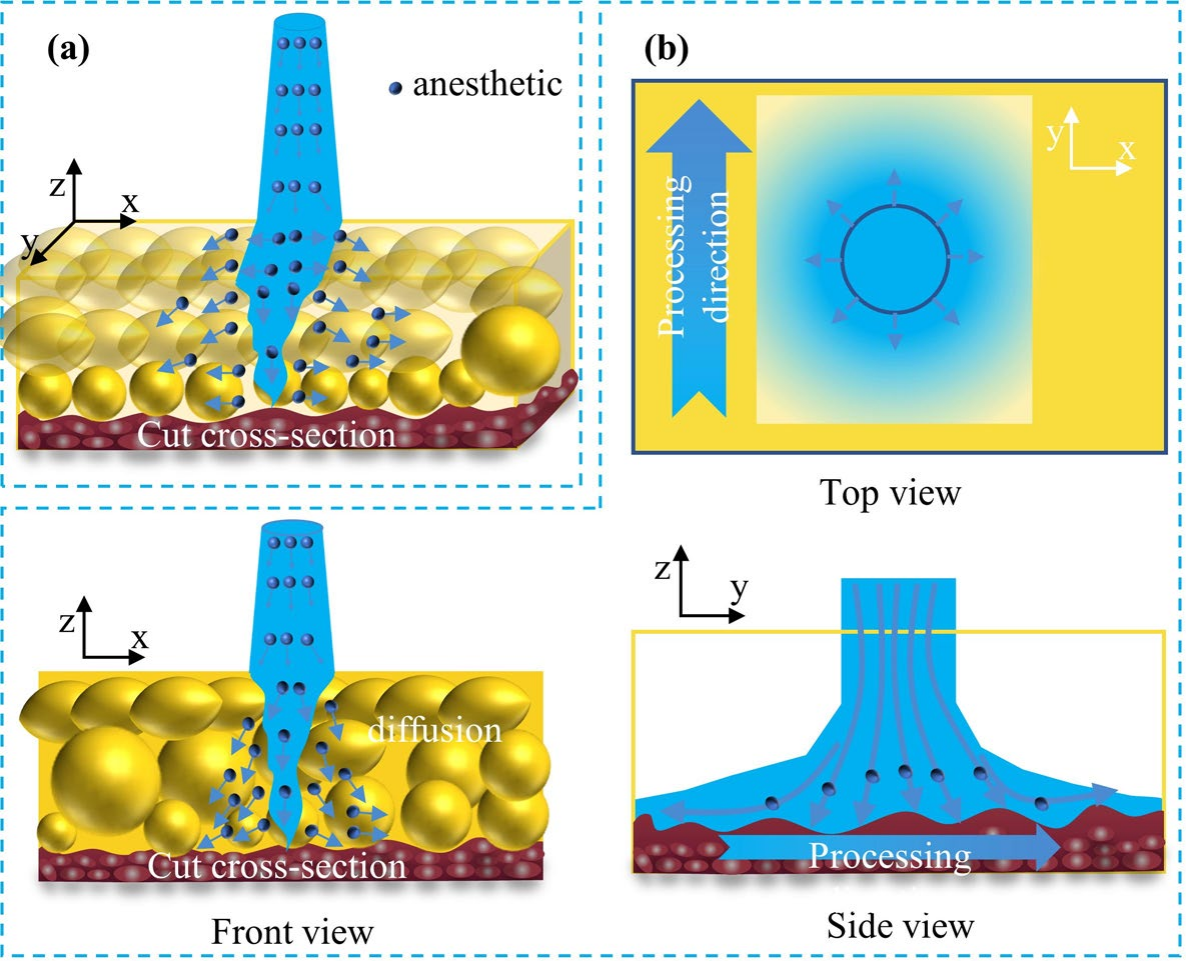
Schematic illustration of the synergistic cutting- infiltration mechanism in soft tissue. (a) Three-dimensional representation of the water jet penetrating the soft tissue; (b) Orthogonal views (Top, Front, and Side) demonstrating the processing direction, cut cross-section, and the radial diffusion of the anesthetic agent into the surrounding tissue.

To address this limitation, we propose an integrated ‘cutting-anesthesia’ strategy. The core mechanism involves pre-mixing the anesthetic agent directly into the jet medium, thereby enabling synchronous drug delivery and infiltration during high-speed tissue dissection (Fig. 1). In contrast to conventional needle infiltration anesthesia, this method leverages the kinetic energy of the jet to drive the drug into interstitial spaces, achieving a uniform, atomized dispersion. This effectively mitigates the risk of localized overdose caused by bolus accumulation in traditional injections^28^. Furthermore, the needle-free operation significantly reduces tissue edema^29^and associated complications arising from mechanical puncture trauma^30^.

To address these research gaps, this study aims to verify the feasibility of integrated cutting-anesthesia synergy and elucidate the effects of the nozzle diameter, jet pressure, and tissue type on system performance. Through comparative analysis with scalpel cuttings, we investigated the mechanisms of water jet-induced histological damage. Photoacoustic imaging is employed to visualize the spatial distribution of anesthetics within tissues. By optimizing the nozzle parameters and jet pressure on the basis of tissue damage and diffusion assessments, this research aims to expand the clinical utility of medical waterjets and support the development of novel jet-based applications.

## Cutting-anesthesia integration technology and experimental platform construction

### Key component

**Fig. 2:**
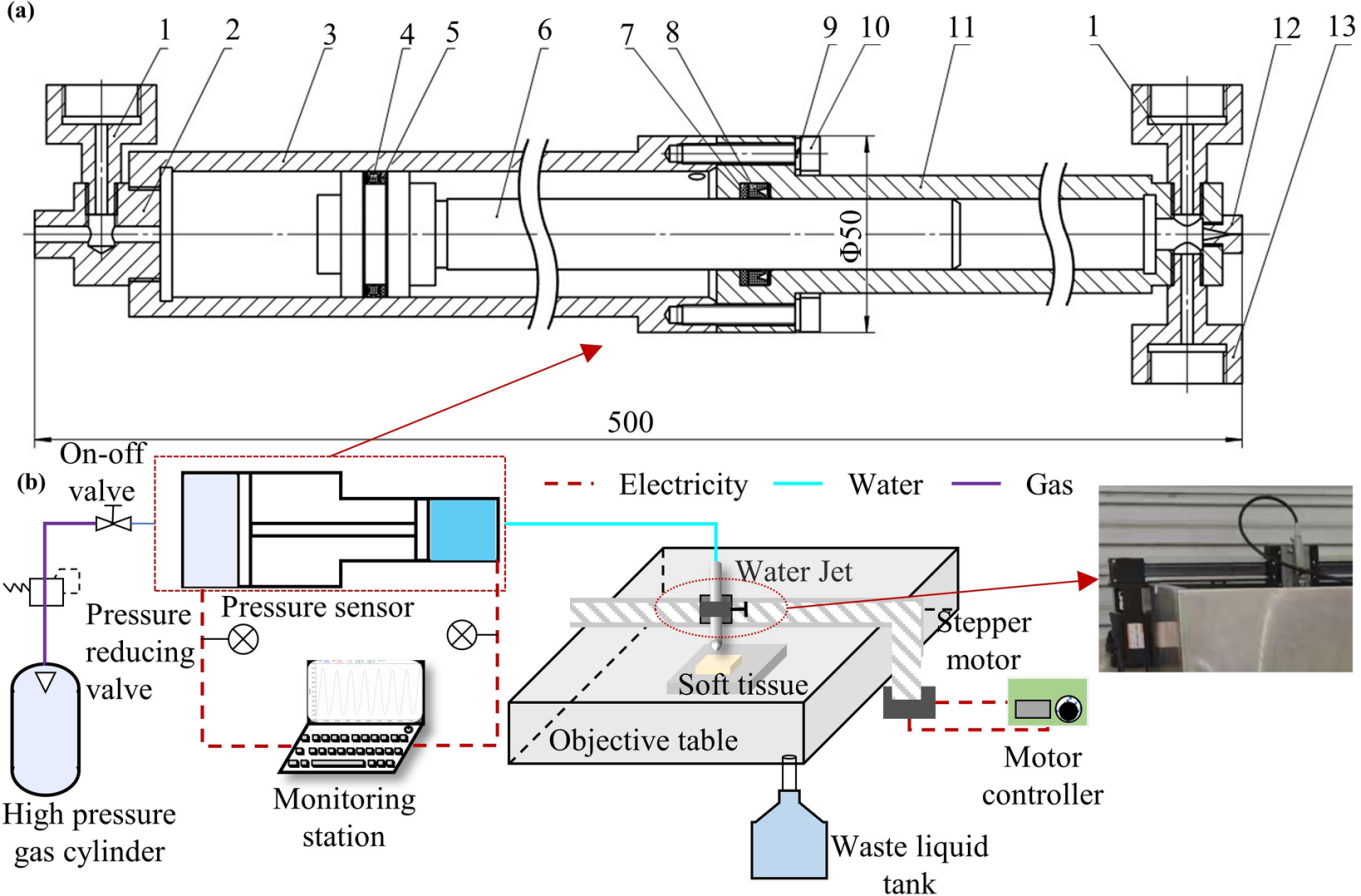
Schematic illustration of the medical integrated water jet system. (a) Detailed cross-sectional view of the core driving component. 1-sensor connector; 2-end cover; 3-rear cylinder body; 4-star seal ring; 5-star seal retaining ring; 6-piston; 7-U-type seal retaining ring; 8-U-type seal ring; 9-spring washer; 10-hexagon socket screw; 11-front cylinder body; 12-nozzle; 13-check valve connector; (b) Overview of the experimental setup.

The integrated water jet is generated using high-pressure gas as the power source to drive a piston, which reciprocates and compresses the medical fluid within a sealed chamber. The pressurized fluid is then accelerated through a nozzle, converting hydraulic pressure into kinetic energy to form a high-speed jet. Leveraging the hydraulic boosting principle, the system achieves high spray pressure with relatively low inlet pressure. The jet generation mechanism is detailed in Fig. 2a.

Piston movement occurs in two distinct stages: the backward stroke and the forward stroke. During the backward stroke, the piston retracts to the correct position where the one-way valve connector opens to the water inlet. The air chamber connects to the high-pressure gas cylinder while the water chamber outlet remains sealed. As high-pressure fluid enters the water chamber, it drives the piston leftward. Upon completion of the backward stroke, the high-pressure gas cylinder switch valve and the water chamber outlet open simultaneously. High-pressure gas then enters the gas chamber, forcing the piston rightward and expelling the fluid from the water chamber to generate a continuous jet. The differential effective areas between the pneumatic and hydraulic chambers create a specific pressure amplification ratio. This design enables the generation of a stable, continuous jet via low-pressure gas input, ensuring both operational safety and cost-effectiveness.

### Experimental platform construction

The experimental platform incorporates a custom-designed variable-area plunger pump featuring synchronized yet independent pneumatic and hydraulic chambers. A continuous stable pressure supply from a high-pressure gas cylinder drives the plunger, generating consistent fluid power that produces a high-speed, stable water jet at the nozzle. The novel variable cross-sectional design achieves pressure amplification ratios of up to 1:3, significantly enhancing the cutting efficiency. The overall structure of the device is shown in Fig. 2b.

To ensure precise control over the cutting process, the water jet nozzle was mounted on a linear automated motion platform to regulate the movement speed. The target distance between the nozzle tip and the tissue surface was set via an adjustable fixture at the nozzle connection. The control and safety systems integrate multiple precision components: a high-precision pressure sensing system (accuracy: 0.1% full-scale) enables real-time dual-chamber pressure monitoring ; the pressure values reported in this study refer to the hydraulic pressure measured directly within the water chamber. Additionally, closed-loop feedback control maintains operational pressure stability. To ensure operational safety, an automated emergency shutdown mechanism is implemented via a safety pressure-reducing valve positioned at the outlet of the high-pressure gas cylinder. When the real-time monitoring system detects that the jet pressure exceeds the preset safety threshold, this valve automatically closes to cut off the pneumatic power source, thereby instantly halting the system operation. These integrated measures effectively prevent hazardous conditions during pressure anomalies.

### Experimental scheme

This study utilized porcine tissue samples comprising two representative soft tissue types: subcutaneous adipose tissue from the abdominal wall and skeletal muscle tissue from the longissimus dorsi muscle. All samples were obtained from healthy adult pigs processed at a licensed abattoir. The interval between slaughter and laboratory processing was strictly controlled within six hours to maintain tissue integrity.

All experimental procedures involving animal-derived tissues were approved by the Institutional Animal Care and Use Committee (IACUC) and conducted in accordance with relevant ethical regulations (Approval No. SYXK2023-0005).

Immediately following collection, samples were placed in pre-cooled containers maintained at 4 °C for cold-chain transport and temporary storage. This protocol effectively minimized post-mortem autolysis, microbial proliferation, and moisture loss, thereby ensuring standardized samples with consistent biomechanical properties for subsequent waterjet cutting experiments.

**Fig. 3:**
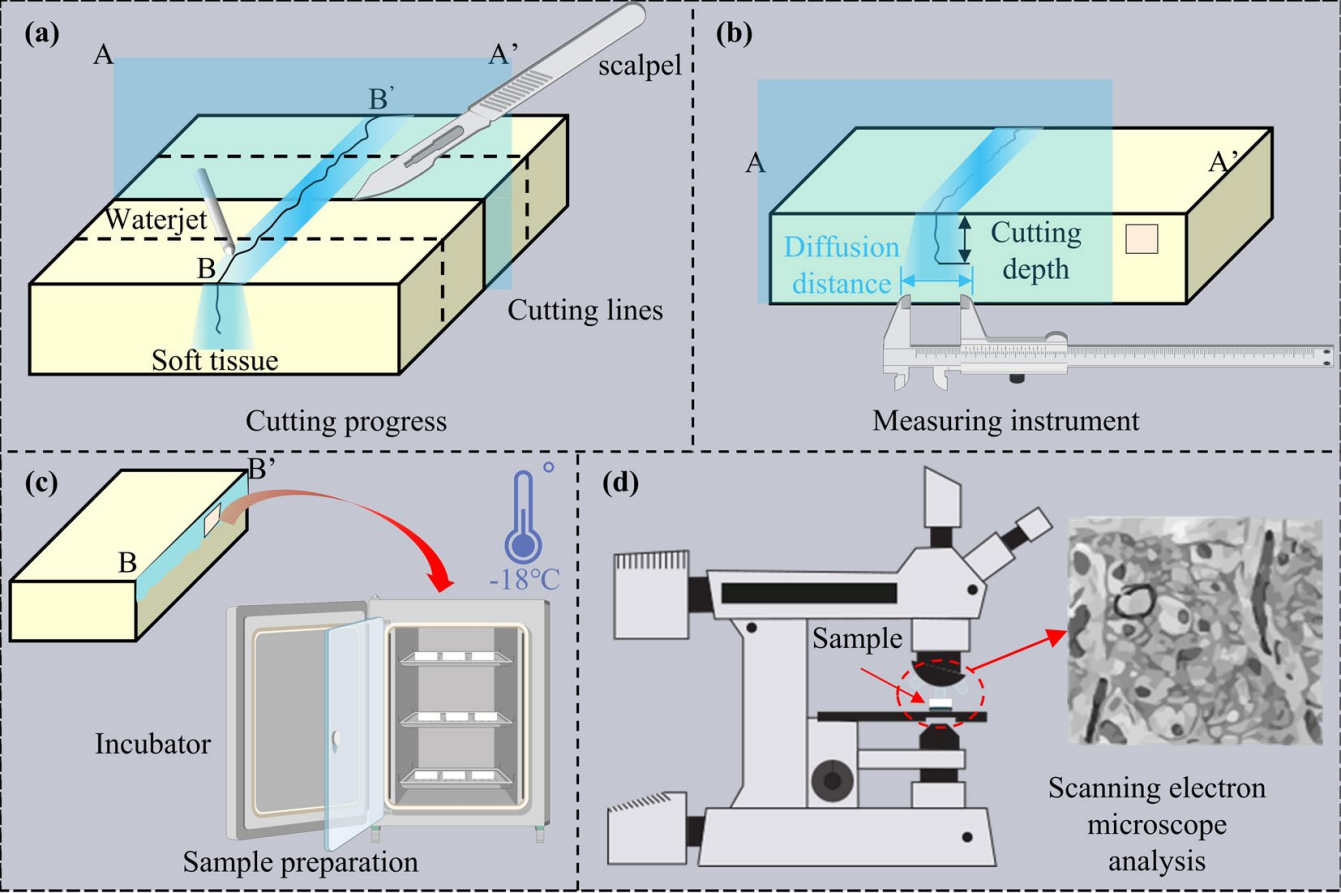
Schematic overview of the experimental workflow and characterization methodology. (a) Surgical procedure; (b) performance quantification; (c) sample preservation; (d) microstructural analysis.

The experimental procedure comprised three distinct parts. In the first part, cutting and diffusion experiments were conducted using a medical integrated water jet (Fig. 3a). The experimental parameters are summarized in Table 1. Fresh porcine tissue, which was sectioned into regular cubic blocks, was securely fixed in a clamp to prevent movement during testing. A methylene blue solution served as the jet medium for soft tissue dissection, with the resulting dyed area interpreted as the drug diffusion region within the tissue^31^. The primary evaluation metrics for this study were the cutting depth and the diffusion range on the tissue cross-section. These parameters were directly quantified using a high-precision vernier caliper (Fig. 3b) by measuring the maximum transverse width (total span) of the methylene blue-stained region.To mitigate experimental randomness, each test condition was replicated three times. This three-replicate experimental approach is also employed in in vitro tissue studies^32–34^, with experimental errors falling within an acceptable range. To minimize the influence of biological heterogeneity inherent in ex vivo tissues, sample preparation was strictly standardized: all porcine tissue samples were harvested from the same anatomical region of the same species and processed under identical temperature and humidity conditions. The results were expressed as mean ± standard deviation.

**Table 1:** Parameters used in the cutting-diffusion experiments.

Nozzle diameter/mm	Pressure/MPa	Target distance/mm	Movement speed/(mm/s)
0.1/0.2/0.3	2/4/6/8/10	5	2.6^36^

The second part of the experiment aimed to evaluate and compare the degree of tissue damage induced by the integrated water jet with that induced by a conventional scalpel. The investigation focused on the microstructure and surface integrity of the incision sites. Three cutting modalities were examined: (1) scalpel, (2) high-pressure water jet (7–8 MPa), and (3) low-pressure water jet (3–4 MPa). Tissue samples were collected at identical depths from incisions made via each method. The samples were preserved at -18 °C (Fig. 3c). To prevent freeze-thaw artifacts, the samples were transferred directly to the cold stage of the scanning electron microscope (SEM) for observation (Fig. 3d). The specimens were maintained in a frozen state throughout the imaging process to ensure structural fidelity. This experimental design resulted in a total of six study groups for comparative analysis (2 tissue types × 3 cutting modalities).

The third phase of the experiment focused on the three-dimensional visualization and quantification of drug diffusion following tissue dissection under optimized waterjet parameters. Informed by the findings from the preliminary cutting-diffusion experiments, the optimal parameters for muscle tissue were selected for this analysis. The jet medium consisted of Indocyanine Green (ICG) dissolved in saline (20 mg/L), which served as a surrogate for the anesthetic agent. Porcine muscle tissue samples were dissected using this ICG-laden solution and immediately subjected to photoacoustic imaging. For quantitative analysis, six data points were sampled at equal intervals for each depth. To mitigate the impact of outliers, the maximum and minimum values were excluded, and the data were reported as mean ± standard deviation.

## Results and discussion

### Effects of the jet parameters on the separation depth and diffusion distance

**Fig. 4:**
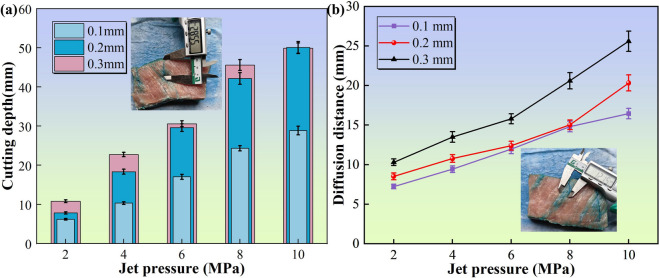
Assessment of tissue dissection capability and anesthetic infiltration in muscle tissue. (a) Influence of jet parameters on incision depth, showing the capability to penetrate muscle tissue under varying pressures; (b) Evaluation of anesthetic diffusion distance, reflecting the extent of drug delivery during the dissection process.

Figure 4 depicts the cutting depth and diffusion distance across varying jet parameters. As shown in Fig. 4a, the cutting depth increases nonlinearly with the jet pressure and is modulated by the nozzle diameter, which is consistent with the findings of Rajesh Ravi et al.^37^. In the low-pressure regime (2–4 MPa), the depth increases rapidly because of kinetic-energy-dominated tissue penetration. Conversely, in the high-pressure range (6–10 MPa), depth escalation attenuates. This attenuation arises from turbulence-induced jet instability (Re > 4000) during jet-tissue interaction. The Reynolds number (Re) quantifies the ratio of inertial to viscous forces to predict flow regimes, and the specific Re values corresponding to each parameter condition are presented in Table 2. Increasing the cutting depth concomitantly expands the standoff distance, causing premature jet diffusion and kinetic energy dissipation in non-target regions. At identical pressures, 0.1 mm nozzles exhibit reduced cutting efficiency, whereas 0.3 mm nozzles achieve maximal depth by transporting greater kinetic energy.

**Table 2:** Reynolds numbers (Re) under different experimental parameter conditions.

Jet pressure/MPa	Reynolds numbers		
	d = 0.1 mm	d = 0.2 mm	d = 0.3 mm
2	6,325	12,649	18,974
4	8,944	17,889	26,833
6	10,954	21,909	32,863
8	12,649	25,298	37,947
10	14,142	28,284	42,426

Figure 4b illustrates the dependence of the diffusion distance on pressure. Unlike the trend observed in cutting depth, the results in Fig. 4b demonstrate a continuous positive correlation between diffusion distance and jet pressure across the tested range, without a distinct saturation plateau. When the pressure is below 4 MPa, the diffusion distance increases with pressure, and the reaction jet exerts a hydraulic splitting effect on the tissue interstitium^38^. Similarly, as the pressure exceeds 4 MPa, the high-pressure fluid continues to expand the diffusion pathways, maintaining the increasing trend in diffusion distance. Additionally, the nozzle diameter has a differentiated impact on the diffusion effect: the 0.1 mm nozzle has the poorest diffusion effect because its energy concentration leads to cutting dominance, whereas the 0.3 mm nozzle has the best cutting effect, reflecting its wide-area penetration characteristics and suitability for scenarios requiring the preservation of surrounding tissues.

**Fig. 5:**
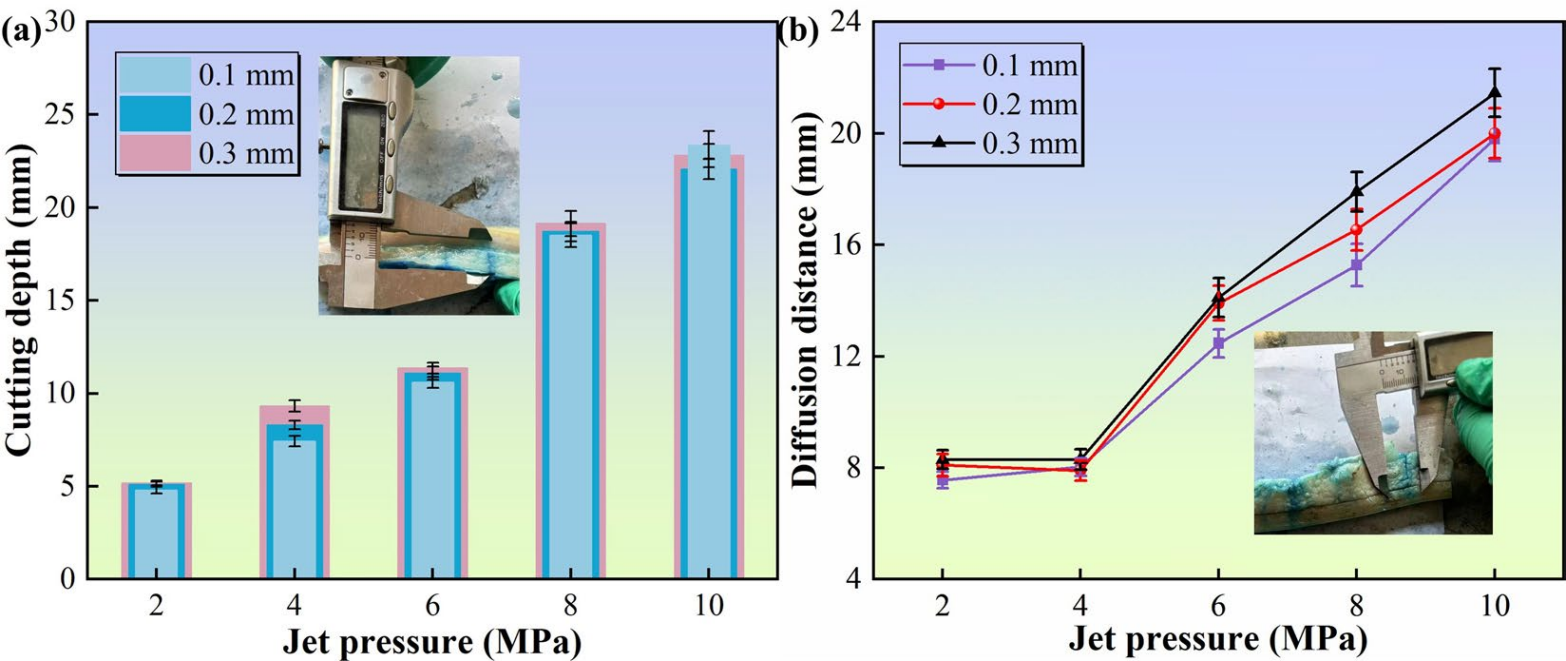
Assessment of tissue dissection capability and anesthetic infiltration in adipose tissue. (a) Influence of jet parameters on incision depth, showing the capability to penetrate adipose tissue under varying pressures; (b) Evaluation of anesthetic diffusion distance, reflecting the extent of drug delivery during the dissection process.

The results of the cutting and diffusion experiments on adipose tissue are presented in Fig. 5. The effects of pressure and nozzle diameter on both the cutting depth and diffusion distance exhibit similar trends. Specifically, the cutting depth of adipose tissue increases more rapidly with pressure, whereas the diffusion distance of the tracer in adipose tissue has a more pronounced dependence on pressure, while the influence of nozzle diameter became less consistent. Under identical pressure conditions, the cutting depth of adipose tissue is lower than that of muscle tissue, and the diffusion distance is also considerably less than that of muscle tissue. This discrepancy can be attributed to the different compositions of the two tissues. Adipose tissue primarily consists of adipose droplets, whereas muscle tissue is predominantly composed of myofibril bundles. The breaking strain of adipose tissue is more than twice that of muscle tissue^39^. Owing to its high lipid content and low mechanical strength, the cutting process of adipose tissue is characterized by “high dissipation-weak diffusion,” which contrasts sharply with the “efficient cutting-controllable diffusion” observed in muscle tissue. These differences arise primarily from the fundamental variations in extracellular matrix density and cellular structural morphology, providing a crucial basis for parameter selection in precision surgery.

The cutting and diffusion experiments conducted on various tissues demonstrated that the integrated water jet effectively achieves a synergistic effect of tissue cutting and targeted drug infiltration, with performance indicators that meet the physical requirements for surgical dissection and delivery. Biological soft tissue, which is primarily composed of cells and an extracellular matrix (ECM), has a complex three-dimensional network structure formed by macromolecules such as collagen fibers, elastic fibers, and reticular fibers^40^. There are marked differences in the content and spatial distribution of these components in different soft tissues^39^. Due to these structural heterogeneity and viscoelastic properties, the cutting depth of the water jet in soft tissue shows a nonlinear positive correlation with the diffusion range of the anesthetic drug. The experimental data indicate that, at the same jet pressure, optimal cutting and diffusion effects are achieved with a nozzle diameter of 0.3 mm, whereas a nozzle diameter of 0.1 mm is the least effective. However, the jet flow rate of the 0.3 mm nozzle is relatively high, which may introduce several issues: the jet medium contains anesthetic components, and an excessive flow rate may lead to drug accumulation in the tissue, thereby increasing the risk of systemic toxicity. Additionally, excessive fluid accumulation can obscure the surgical field and interfere with the surgeon’s operations. Furthermore, higher jet pressures yield better outcomes, necessitating optimization according to clinical requirements.

In this study, the definition of ‘optimal’ parameters was not based solely on maximizing a single physical magnitude, but rather on achieving a balance between surgical efficiency and safety. We established a two-tier criterion for parameter selection: The primary requirement was to achieve a stable cutting depth of at least 20 mm, which is clinically essential for effective dissection of parenchymal organs. Under the premise of meeting the depth requirement, the objective was to minimize collateral tissue damage and prevent uncontrolled fluid saturation.

Our experimental results indicated that the 4 MPa pressure with a 0.2 mm nozzle configuration successfully met the 20 mm depth threshold^41^. While higher pressures (> 4 MPa) yielded deeper incisions, they resulted in disproportionately higher collateral damage and excessive fluid diffusion, as evidenced by the histological analysis. Conversely, pressures below 4 MPa failed to consistently penetrate to the required depth. Therefore, the 4 MPa/0.2 mm combination was identified as the optimal operating point that maximizes therapeutic efficacy while maintaining safety standards.

It is important to validate the use of Methylene Blue and ICG as surrogates for clinical anesthetics. Hydrodynamically, calculations based on our jet parameters indicate a Re range of 6325–42,426, placing the flow firmly in the fully turbulent regime (Re > 4000). In this regime, inertial forces dominate viscous forces, meaning the macroscopic jet structure and penetration are governed by the kinetic energy and density of the solvent rather than minor rheological variations of the trace solutes. Physiochemically, the extremely low mass fraction (0.002%) of the dyes ensures the solution retains Newtonian behavior virtually identical to water. Furthermore, regarding diffusion kinetics, the molecular weight of ICG (775 g/mol) is significantly higher than that of common local anesthetics. Since the diffusion coefficient is inversely proportional to molecular size, the diffusion range observed in this study represents a conservative estimation, implying that clinical anesthetics would theoretically achieve equal or more extensive tissue coverage.

### Selective cutting with an integrated water jet

**Fig. 6:**
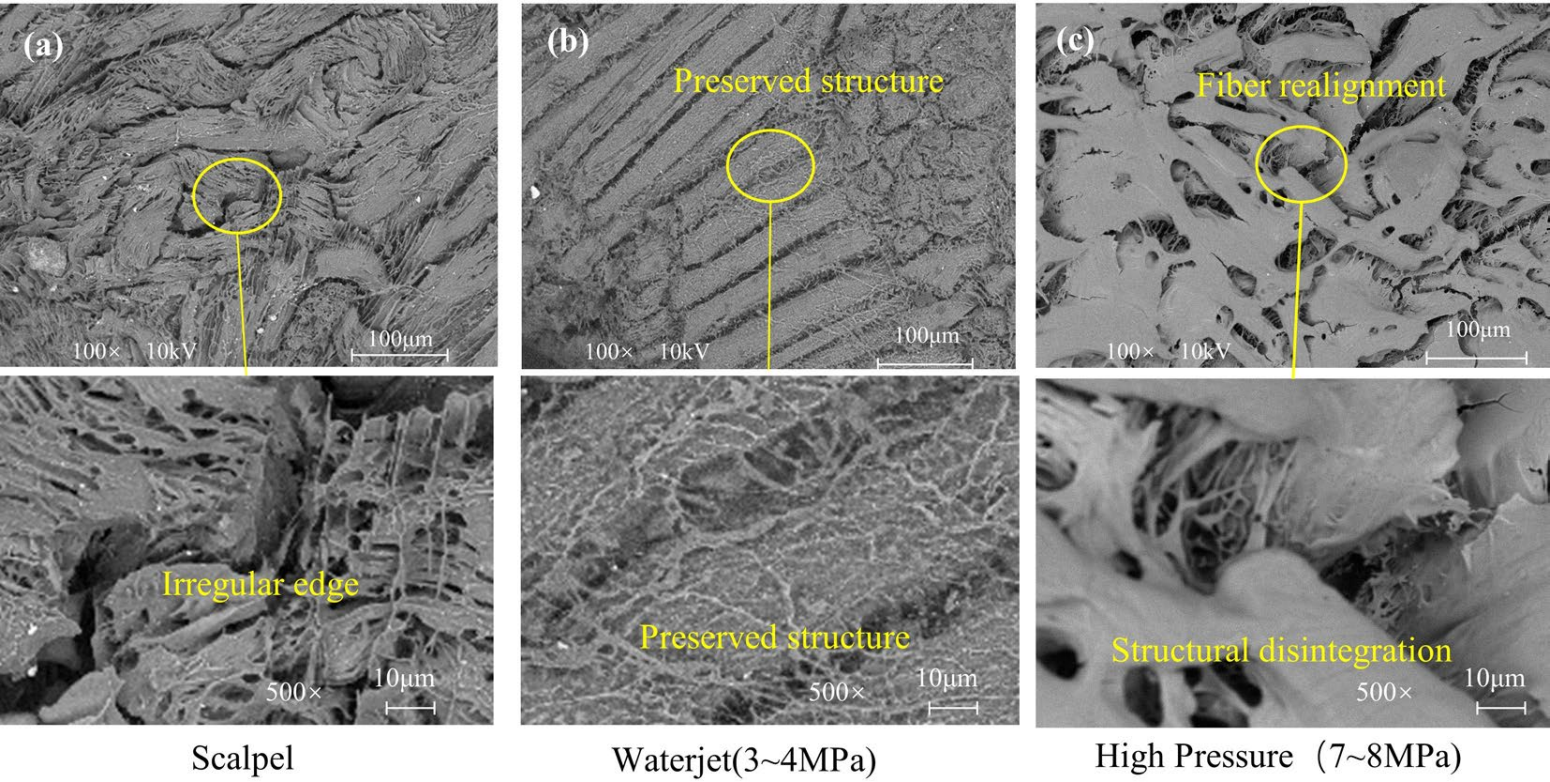
SEM micrographs comparing the muscle tissue incision surface morphology under different cutting conditions. (a) Conventional scalpel cutting; (b) low-pressure waterjet cutting (3–4 MPa); (c) high-pressure waterjet cutting (7–8 MPa).

On the basis of the critical cutting parameters identified above, tissue damage was observed in two types of tissue under scalpel cutting conditions at 3–4 MPa and 7–8 MPa. In this study, tissue sections 10 mm beneath the surface were analyzed, and soft tissue incision morphology under different parameters was examined by SEM. Prior to evaluating the surgical damage, we assessed the potential impact of the preservation process. While freezing without cryoprotection can theoretically induce microstructural voids^42^, our cold-stage SEM observation revealed no evidence of such artifacts. The Low-Pressure group (Fig. 6b) exhibited a dense and continuous tissue structure without the formation of large freezing-induced voids. Consequently, the specific tissue damage forms analyzed below are attributed to the distinct mechanical effects of the surgical modalities rather than preservation artifacts.

The SEM results for muscle tissue are shown in Fig. 6. The image following scalpel cutting (Fig. 6a) reveals irregular fractures and jagged edges in the muscle fibers, indicating that traditional mechanical cutting causes tearing damage, which may adversely affect postoperative healing^43^. In contrast, the low-pressure water jet group (Fig. 6b) exhibited relatively orderly muscle fiber alignment in parallel strips, with distinct boundaries between fiber bundles and partial preservation of the overall tissue architecture. High-magnification images further revealed structurally intact connective tissue within the fibrous matrix. Conversely, the high-pressure water jet group (Fig. 6c) exhibited extensive fiber rupture, structural disintegration, and compromised muscle integrity, suggesting that such damage markedly impaired functional recovery.

**Fig. 7:**
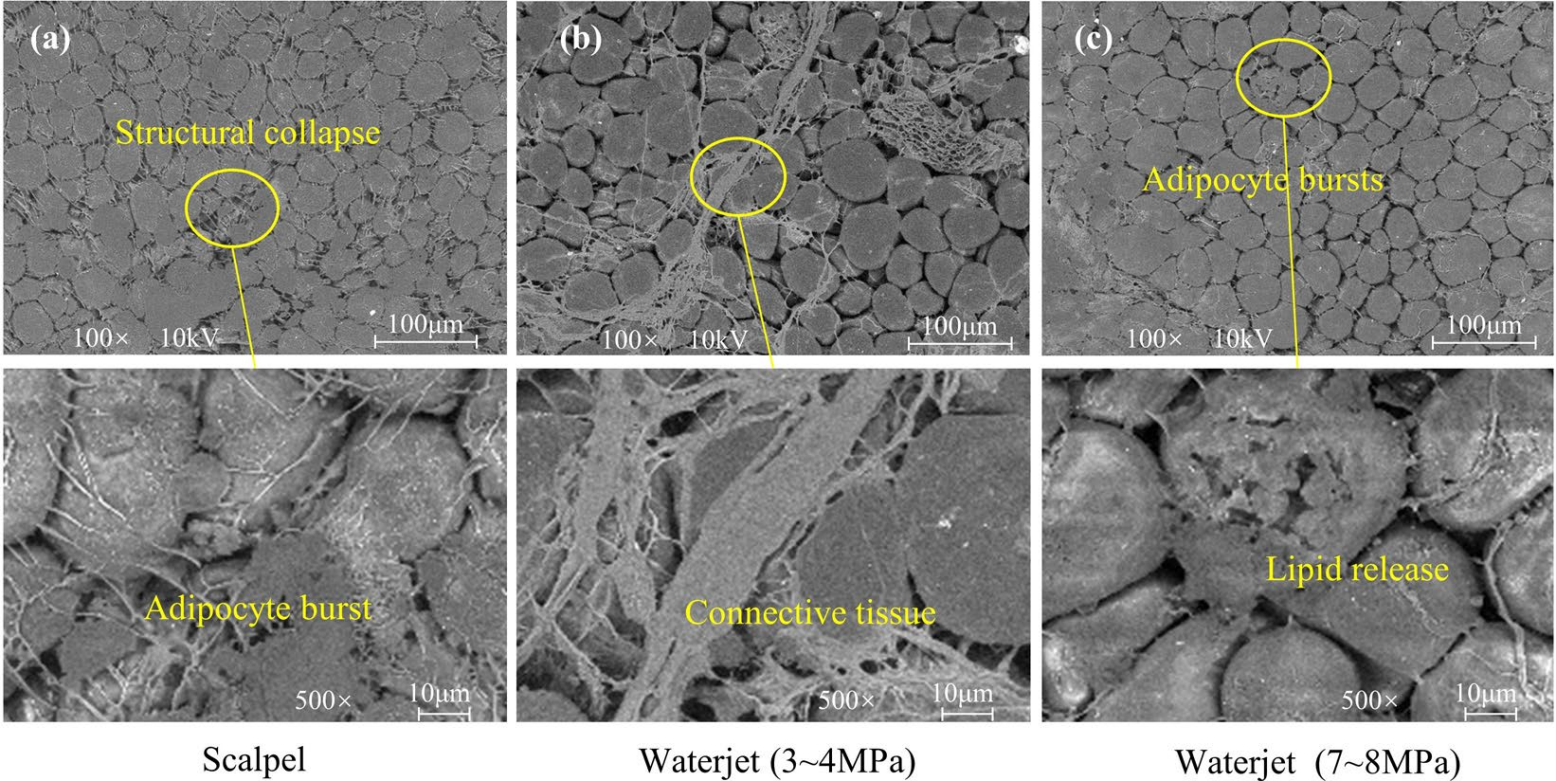
SEM micrographs comparing the incision surface morphology of adipose tissue under different cutting conditions. (a) Conventional scalpel cutting; (b) low-pressure waterjet cutting (3–4 MPa); (c) high-pressure waterjet cutting (7–8 MPa).

Under low-magnification scanning electron microscopy (Fig. 7a), adipose tissue exhibited reduced intercellular spaces, attributed to the compressive forces generated during scalpel-based mechanical cutting, which induced tissue deformation^44^. This deformation results in denser cellular packing and diminished extracellular gaps. High-magnification imaging further revealed signs of apoptosis and microstructural disruption, including rupture of intracellular contents. In the low-pressure water jet group (Fig. 7b), SEM analysis revealed that although slight tissue expansion occurred due to fluid infiltration, the integrity of the connective tissue was largely preserved, with minimal adipocyte rupture. High-magnification images clearly revealed a continuous connective tissue network supporting the adipocytes, with preserved cell morphology and structural integrity. In contrast, the high-pressure water jet group (Fig. 7c) presented significant adipocyte compression and a notable decrease in the intercellular space. Although no widespread structural damage was observed, the localized cellular architecture was severely compromised, indicating that high-pressure fluid penetration may cause mechanical disruption of the cell membrane.

**Fig. 8:**
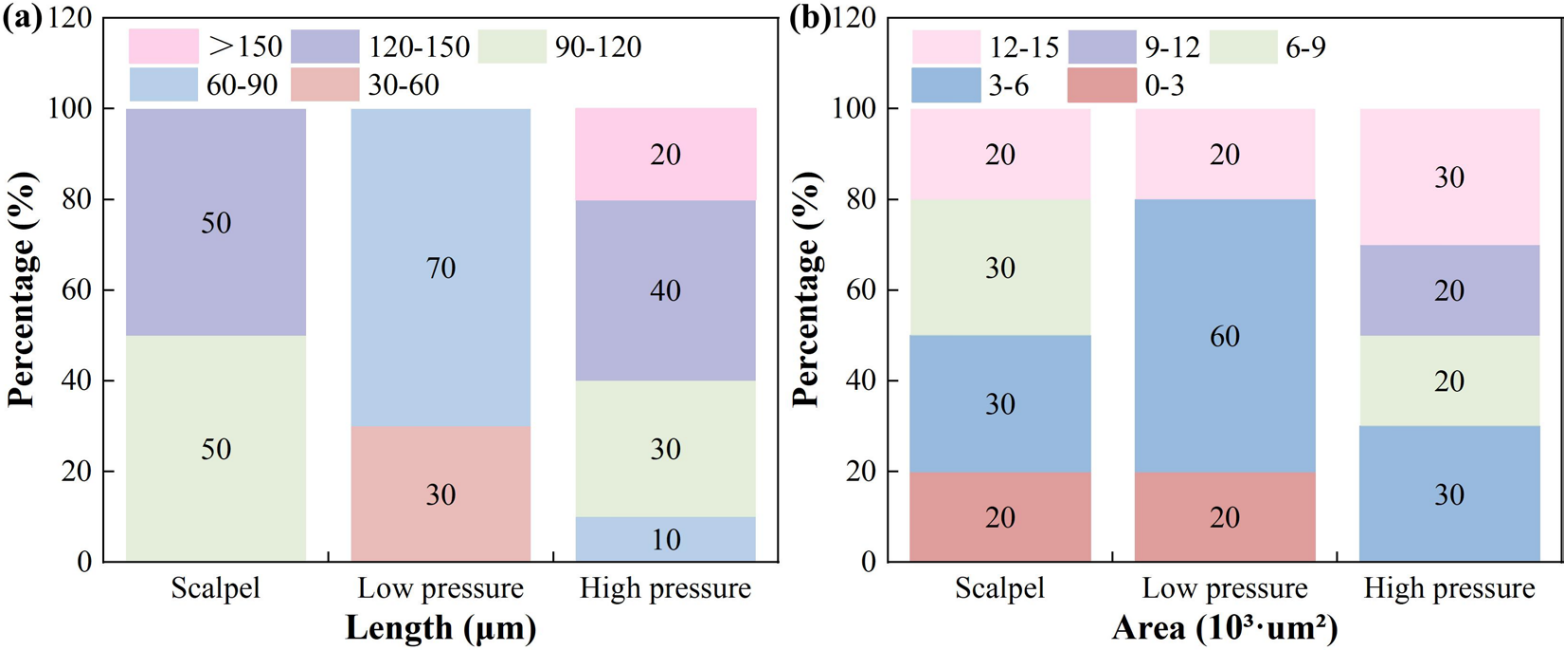
Statistical distribution of tissue micro-damage dimensions under different cutting modalities. (a) Distribution of muscle fiber fracture lengths; (b) distribution of adipose tissue damage areas.

Muscle fiber break length is a critical parameter for evaluating tissue damage and predicting wound healing time. In this study, we quantified muscle fiber break lengths under three treatment conditions (Fig. 8a). In the high-pressure water jet group (7–8 MPa), the fracture lengths predominantly ranged from 90 to 150 μm, with some exceeding 150 μm—significantly longer than those in the other groups. This can be attributed to the high dynamic impact pressure and turbulence-induced shear stresses, which caused extensive tissue disruption rather than clean incisions. In contrast, the low-pressure water jet group (3–4 MPa) exhibited break lengths primarily between 30 and 90 μm, with preservation of functional structures (intact tissue width: 39.45 μm), indicating a more controlled extent of damage. These findings suggest that the low-pressure water jet achieves hydrodynamic dissection mainly through fluid shear, facilitating tissue separation while minimizing collateral damage and demonstrating a degree of tissue selectivity. The scalpel group presented a relatively concentrated distribution of fracture lengths (90–150 μm), which partially overlapped with those of the high-pressure group. Scalpel cutting, governed by mechanical shear, typically produces sharp, localized incisions rather than the large-scale observed with fluid-based methods.

The extent of cellular damage is a critical factor influencing wound healing outcomes. In this study, we quantitatively evaluated adipose tissue damage areas under three treatment modalities (Fig. 8b). Comparative analysis revealed distinct intergroup differences in both damage distribution and severity. The scalpel group exhibited a broad yet relatively uniform damage pattern, with high-damage regions (12–15 × 10^3^ µm^2^) comprising 20% of the total area. This reflects typical mechanical cutting failure, where concentrated shear forces produce localized damage with well-defined margins. In contrast, the low-pressure water jet group exhibited a predominant damage area of 3–6 × 10^3^ µm^2^, accounting for 60% of the total area, with high-damage regions also representing 20% of the total area. These results indicate that low-pressure hydrodissection enables controlled tissue separation while minimizing collateral injury, demonstrating its selective tissue-preserving effect. The high-pressure water jet group exhibited the most extensive tissue disruption, with the total damaged area (across all severity levels) being 1.5 times greater than that of the scalpel group and twice that of the low-pressure group, which is consistent with the findings of Sidorov et al.^45^. Notably, high-damage regions (12–15 × 10³ µm²) alone accounted for 30% of the affected area—highest among all the groups. This pronounced tissue damage suggests that when the jet pressure exceeds the elastic threshold of the tissue, residual kinetic energy leads to widespread structural compromise beyond the intended dissection zone.

The choice of tissue separation technique significantly influences surgical outcomes. Traditional scalpel dissection can lead to adverse effects due to mechanical shear forces, including disruption of cell membrane integrity, tearing of muscle fibers, and exacerbation of secondary inflammatory responses. This study demonstrated that low-pressure water jet technology (3–4 MPa) offers distinct advantages. Tissue separation via fluid shear selectively preserves the connective tissue framework, significantly reduces functional tissue damage (with a 35% reduction in the damaged area), and facilitates postoperative functional recovery. However, when the jet pressure exceeds the critical threshold (> 7 MPa), negative outcomes are observed, including compromised adipocyte structure (with a 35% increase in damaged area), destruction of the muscle fiber network (with rupture lengths exceeding 120 μm), and overall tissue injury surpassing that caused by mechanical cutting. These findings highlight the importance of precise jet parameter control tailored to surgical objectives. Recommendations for clinical application include: (1) using a low-pressure mode (3–4 MPa) for delicate procedures, such as plastic surgery; (2) applying a medium-pressure mode (5–6 MPa) for routine operations; and (3) reserving a high-pressure mode (7–8 MPa) for extensive resections requiring aggressive tissue removal.

### Three-dimensional diffusion mechanism of anesthetics in muscles

**Fig. 9:**
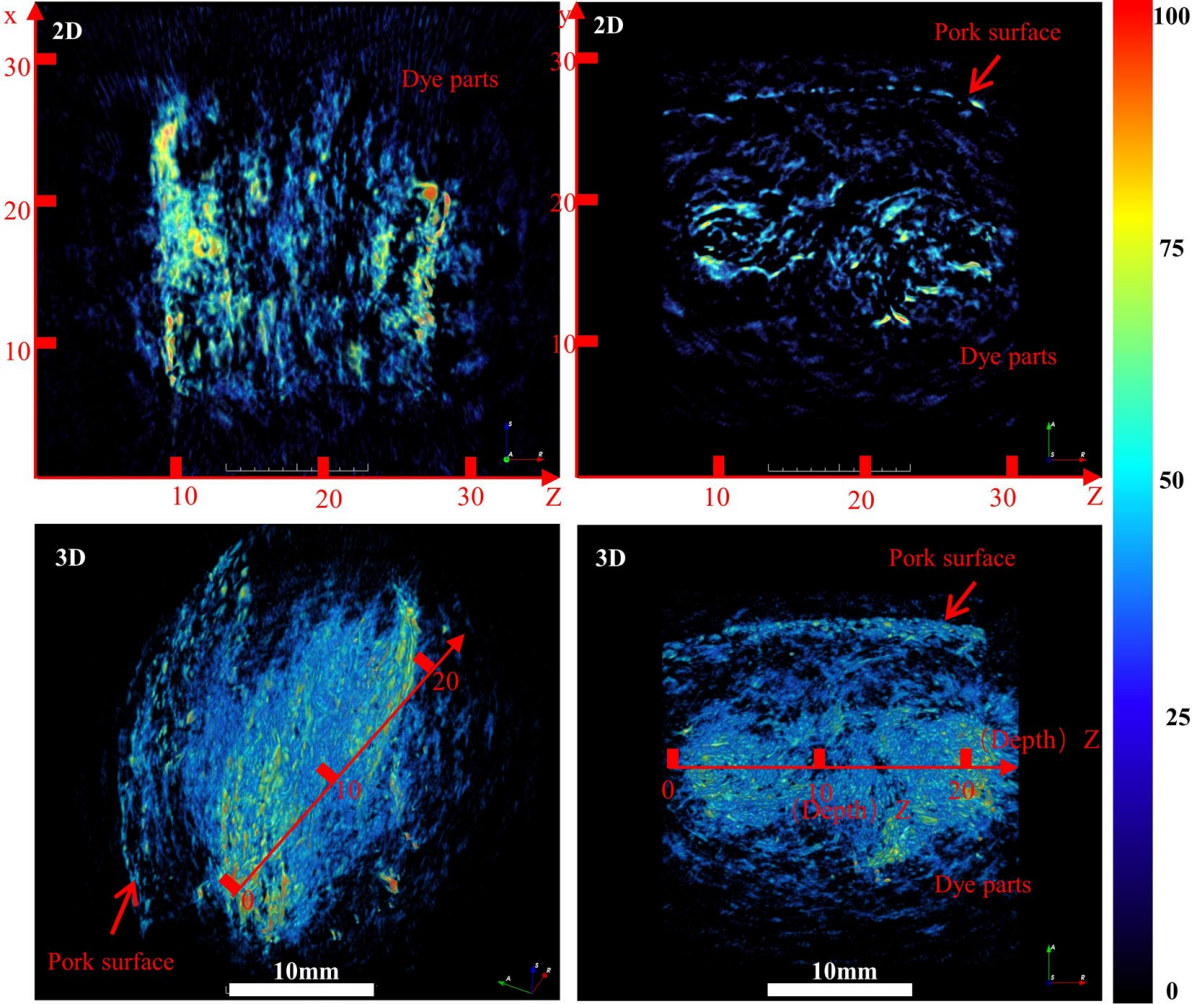
Volumetric visualization of the spatial distribution of anesthetic dye in porcine muscle tissue.

Building on previous findings that muscle tissue exhibits pronounced drug diffusion characteristics, this study explored the spatial distribution of anesthetic agents under critical cutting parameter conditions. As the spatial distribution and saturation volume of therapeutic agents within tissues are critical determinants for drug delivery efficiency, we employed photoacoustic imaging to visualize the 3D diffusion behavior of anesthetics in muscle tissue (Fig. 9). Two-dimensional imaging revealed a distinct annular high-concentration zone near the surface, along with a localized deep-region hotspot, likely resulting from prolonged drug accumulation and hydrodynamic penetration. Three-dimensional imaging further demonstrated an exponential decay in drug concentration with increasing tissue depth, which was consistent with the modified Fick’s diffusion law. Notably, the diffusion front exhibited an irregular, fractal-like pattern, indicating significant anisotropic diffusion—potentially influenced by structural barriers such as the perimysium. This study is the first to characterize complex anesthetic diffusion patterns in biological tissues via 3D imaging, providing a critical theoretical basis for optimizing hydrodynamic delivery parameters. these findings underscore the importance of accounting for tissue structural heterogeneity in clinical anesthesia and support the development of depth-specific dosing protocols to maximize tissue saturation and potential nerve coverage.

**Fig. 10:**
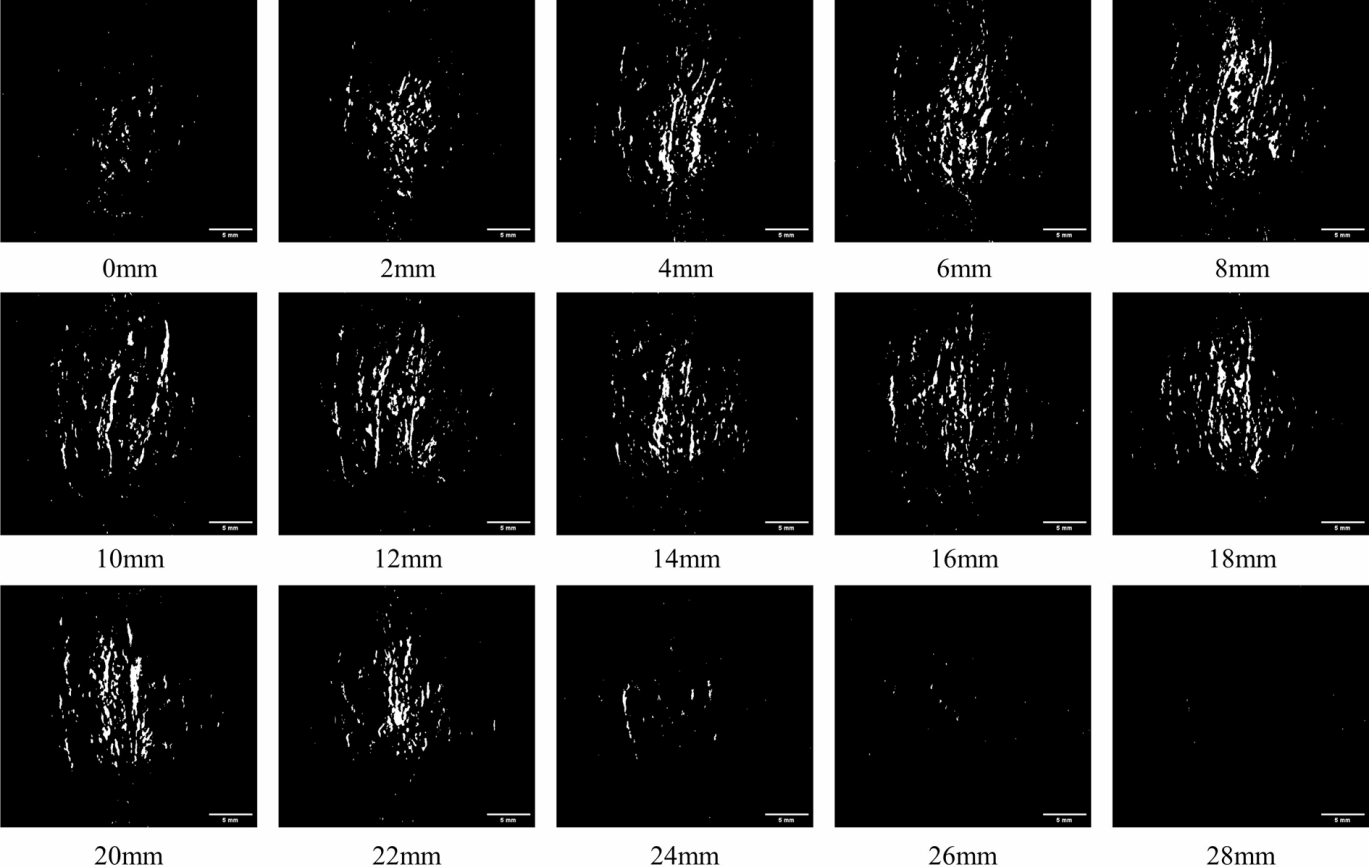
Sequential cross-sectional images illustrating the anesthetic diffusion distribution at varying depths (0–28 mm). The images display the drug infiltration pattern from the surface downwards at 2 mm intervals.

To characterize the diffusion characteristics of drugs in tissues quantitatively, this study employed a systematic stratified sampling method to obtain tissue sections from the surface to the deep layer of the cut surface at intervals of 2 mm, facilitating the analysis of diffusion distribution (Fig. 10). Analysis of the depth-dependent diffusion characteristics revealed that in the shallow and middle layers (0–20 mm), the range of drug diffusion exhibited a notably positive correlation with tissue depth. However, in the deep layers (greater than 20 mm), the diffusion range experienced marked attenuation. This phenomenon can be elucidated through principles of fluid dynamics: in the superficial regions of the tissue, the jet sustains a higher initial pressure, coupled with an extended penetration time. Driven by the pronounced pressure gradient, drug molecules can achieve greater diffusion depths. However, as the tissue depth increases, the jet pressure decays exponentially, leading to a sharp reduction in the driving pressure difference within the deeper regions, which subsequently results in a notable decrease in the diffusion efficiency. Furthermore, the morphological characteristics of the diffusion boundary indicate that in the shallow region, the diffusion boundary resembles an irregular tree-like fractal, suggesting that the solution preferentially diffuses along tissue gaps and fiber bundles. Conversely, in the deeper region, the diffusion area evolves into discrete spots, indicating that the solution primarily undergoes restricted percolation through localized microchannels.

**Fig. 11:**
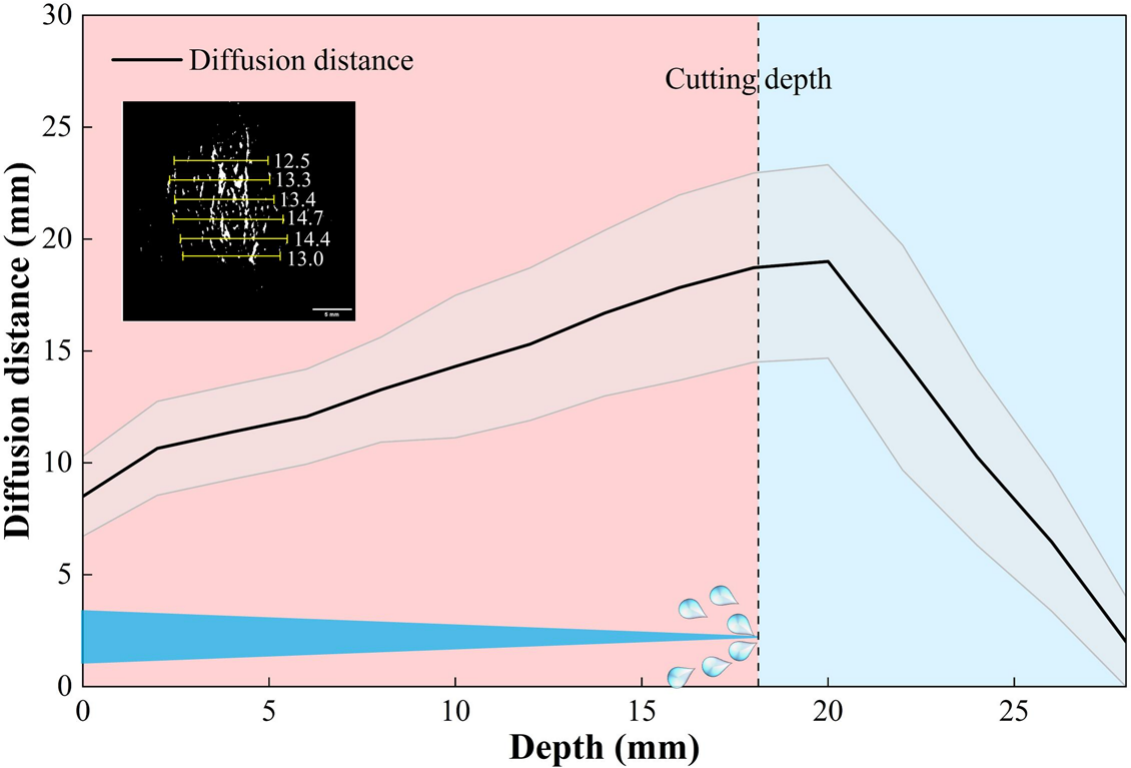
Variation trend of drug diffusion distribution distance with tissue depth. The solid line represents the mean diffusion distance, and the shaded area represents the standard deviation (SD). Data were calculated from 6 equidistant points per depth after excluding the maximum and minimum values to ensure statistical robustness.

Figure 11 illustrates the systematic quantitative analysis of the relationship between drug diffusion distance and tissue depth. The solid curve represents the mean diffusion distance derived from the trimmed dataset, while the shaded region indicates the standard deviation (SD), reflecting the stability of the experimental data. The results demonstrate that drug diffusion behavior exhibits distinct depth-dependent characteristics. Specifically, in the region corresponding to the cutting depth (0–18.31 mm), the mean diffusion distance shows a notable positive correlation with tissue depth. The relatively narrow error band in this region indicates high repeatability of the hydrodynamic injection process. This observed increase is attributed to the hydrodynamic accumulation of incision fluid within the cavity and the expansion of intercellular spaces resulting from cellular relaxation following jet erosion.

The diffusion distance peaks near the cutting boundary (18.31 ± 2 mm) before exhibiting an exponential decay in the deeper layers. In contrast to the eroded upper layers, the tissue beyond the cutting depth experiences minimal mechanical damage and retains a greater cytoplasmic density with intact structural integrity. These factors act as a natural barrier, limiting passive permeation and collectively contributing to the rapid attenuation of the diffusion distance observed in the deep zones.

It is important to acknowledge that this study was conducted using an ex vivo porcine model. Although the hydrodynamic results confirm the efficient physical delivery and extensive infiltration of the surrogate tracers, this model cannot evaluate physiological anesthesia effects, drug metabolism, or the functional duration of analgesic action due to the absence of blood circulation and nerve conduction. The current findings serve as a physical engineering validation, verifying the device’s capability to deliver sufficient fluid volume to the target depth. Future in vivo studies will be prioritized to assess the pharmacokinetic profile and functional pain mitigation in a living system.

## Conclusion

This study successfully developed an integrated cutting-infiltration water jet system based on hydrodynamic principles, enabling synchronous tissue dissection and localized drug delivery. By incorporating anesthetic agents directly into the jet stream, the system achieves dual functionality—precise tissue separation and simultaneous fluid infiltration—while significantly reducing mechanical and structural trauma compared with conventional methods. The key findings are summarized as follows.

1. The cutting-diffusion experiment systematically validated the synergistic effect between tissue cutting and fluid infiltration, revealing a nonlinear positive correlation between the geometric characteristics/jet parameters of the nozzle and both the depth of tissue cutting and the drug diffusion distance. Systematic parametric evaluation revealed the optimal operational parameters for different tissue types: muscle tissue (jet pressure: 4 MPa, nozzle diameter: 0.2 mm), and adipose tissue (jet pressure: 8 MPa, nozzle diameter: 0.2 mm).
2. Microstructural analysis revealed that, compared with conventional scalpel excision, the water jet cutting technique has improved tissue selectivity. Quantitative histological analysis revealed that water jet cutting reduced tissue fiber breakage by 51%, decreased the area of cellular damage by 35%, and maximally preserved 39.45 μm of functional tissue architecture.
3. Photoacoustic imaging confirmed the “cutting-guided diffusion” mechanism, demonstrating that the anesthetic agent exhibited nonmonotonic diffusion dynamics, with the maximum diffusion distance occurring in the vicinity of the cutting depth (18.31 ± 2 mm).

Collectively, these findings establish a foundational framework for device–drug synergy in minimally invasive surgery. By integrating hydrodynamic cutting with targeted drug delivery, this system overcomes key limitations of conventional methods—such as inconsistent drug distribution and collateral tissue damage—while enabling precise therapeutic delivery precisely where dissection occurs.

It should be noted that this study was conducted using an ex vivo model. While the hydrodynamic results confirm the efficient physical delivery of the anesthetic agent, this model cannot evaluate physiological anesthesia effects, drug metabolism, or the duration of analgesic action due to the absence of blood circulation and nerve conduction. The current findings serve as a physical engineering validation, verifying the device’s capability to deliver sufficient fluid volume to the target depth. Future in vivo studies will be prioritized to assess the pharmacokinetic profile, effective concentration thresholds, and functional pain mitigation in a living system.

## Data Availability

The data that support the findings of this study are available from thecorresponding author upon reasonable request.

## References

[1] Hajilo, P., Imani, B., Zandi, S. & Mehrafshan, A. Comparing the intraoperative and postoperative complications of the scalpel and electrocautery techniques for severing the inner layers of the lumbar disc during discectomy surgery. *Front. Surg.* **10**, 1264519 (2023). 37841816 10.3389/fsurg.2023.1264519 PMC10568066

[2] Elmore, L. et al. Evaluating the healing potential of J-plasma scalpel-created surgical incisions in Porcine and rat models. *Biomedicines* **12**, 277 (2024). 38397879 10.3390/biomedicines12020277 PMC10886613

[3] Barati, B. et al. Scalpel versus electrocautery skin incisions in postauricular tympanoplasty: a randomized clinical trial study. *Egypt. J. Otolaryngol.* **41**, 105 (2025).

[4] Hreha, P. et al. Water jet technology used in medicine. *Tehnicki Vjesn.* **17**, 237–240 (2010).

[5] Papachristou, D. N. & Barters, R. Resection of the liver with a water jet. *Br. J. Surg.* **69**, 93–94 (1982). 7059775 10.1002/bjs.1800690212

[6] Hata, Y. et al. Liver resection in children, using a water-jet. *J. Pediatr. Surg.* **29**, 648–650 (1994). 8035275 10.1016/0022-3468(94)90732-3

[7] Une, Y. et al. Liver resection using a water jet. *Cancer Chemother. Pharmacol.* **23**, 74–S77 (1989). 10.1007/BF00647245 2924388

[8] Izumi, R. et al. Hepatic resection using a water jet dissector. *Surg. Today*. **23**, 31–35 (1993). 8384906 10.1007/BF00308996

[9] Rau, H., Duessel, A. & Wurzbacher, S. The use of water-jet dissection in open and laparoscopic liver resection. *HPB* **10**, 275–280 (2008). 18773110 10.1080/13651820802167706 PMC2518306

[10] Gilling, P. J. et al. Five-year outcomes for aquablation therapy compared to TURP: results from a double-blind, randomized trial in men with LUTS due to BPH. *Can. J. Urol.* **29**, 10960–10968 (2022). 35150215

[11] Elterman, D. et al. Meta-analysis with individual data of functional outcomes following aquablation for lower urinary tract symptoms due to BPH in various prostate anatomies. *BMJ Surg. Interventions Health Technol.* **3**, e000090 (2021). 10.1136/bmjsit-2021-000090 PMC8749268 35047807

[12] Nguyen, D. D. et al. Waterjet ablation therapy for endoscopic resection of prostate tissue trial (WATER) vs WATER II: comparing aquablation therapy for benign prostatic hyperplasia in 30–80 and 80–150 mL prostates. *BJU Int.* **125**, 112–122 (2020). 31599044 10.1111/bju.14917 PMC6972548

[13] Cecinato, P. et al. Endoscopic submucosal dissection in colorectal neoplasia performed with a waterjet system-assisted knife: higher en-bloc resection rate than conventional technique. *Clin. Endoscopy*. **55**, 775–783 (2022). 10.5946/ce.2022.099 PMC9726436 36464827

[14] Kotecha, K. et al. Waterjet pulse lavage as a safe adjunct to video assisted retroperitoneal debridement in necrotising pancreatitis. *Surg. Endosc.* **38**, 6973–6979 (2024). 39367136 10.1007/s00464-024-11297-6 PMC11525386

[15] Wormald, J. C., Wade, R. G., Dunne, J. A., Collins, D. P. & Jain, A. Hydrosurgical debridement versus conventional surgical debridement for acute partial-thickness burns. *Cochrane Database Syst. Rev.* (2020). 10.1002/14651858.CD012826.pub2 PMC8094409 32882071

[16] Shimada, K., Ojima, Y., Ida, Y. & Matsumura, H. Efficacy of Versajet hydrosurgery system in chronic wounds: a systematic review. *Int. Wound J.* **18**, 269–278 (2021). 33759367 10.1111/iwj.13528 PMC8244081

[17] Xia, L. et al. A retrospective cohort study comparing the clinical outcomes of the hydrosurgery system and traditional Single-Incision surgery for axillary osmidrosis. *J. Cosmet. Dermatol.* **24**, e16755 (2025). 39801371 10.1111/jocd.16755 PMC11726132

[18] Moon, B. & Kim, Y. J. Comparison of effectiveness and associated complications between conventional subdermal excision and hydrosurgery (VersajetTM) for osmidrosis. *Aesthetic Plast. Surg.* **45**, 3029–3036 (2021). 34351507 10.1007/s00266-021-02485-z

[19] Azab, M. A., Sarhan, K., Atallah, O., Kammoun, B. & Hazim, A. Slicing through complexity: a systematic review assessing the efficacy and safety of water-jet dissection in neurosurgical procedures with technical suggestions. *Neurosurg. Rev.* **48**, 432 (2025). 40397185 10.1007/s10143-025-03568-0

[20] Zhao, J., Song, X. F., Wei, X., Yu, W. & Jing, X. Non-rigid cutting characteristics and separation mechanisms of soft muscle tissue under waterjet impact. *Proc. Institution Mech. Eng. Part. H: J. Eng. Med.* **239**, 485–497 (2025). 10.1177/09544119251333679 40298135

[21] Babaiasl, M. et al. Predictive mechanics-based model for depth of cut (DOC) of waterjet in soft tissue for waterjet-assisted medical applications. *Med. Biol. Eng. Comput.* **58**, 1845–1872 (2020). 32514828 10.1007/s11517-020-02182-0

[22] Srivastava, A., Kundu, A. & Paul, A. R. A detailed review of the recent development of needle-free drug delivery devices. *J. Med. Eng. Technol.* **2025**, 1–20 (2025). 10.1080/03091902.2025.2508893 40418150

[23] Zhu, Y., Kang, C., Cai, W. & Huang, C. Drug injection and dispersion characteristics of an air-powered needle-free injector. *Med. Eng. Phys.* **109**, 103906 (2022). 36371083 10.1016/j.medengphy.2022.103906

[24] Jing, Y., Magnin, I. E. & Frindel, C. Monte Carlo simulation of water diffusion through cardiac tissue models. *Med. Eng. Phys.* **120**, 104013 (2023). 37673779 10.1016/j.medengphy.2023.104013

[25] Caine, M. et al. In situ evaluation of Spatiotemporal distribution of doxorubicin from Drug-eluting beads in a tissue mimicking Phantom. *Eur. J. Pharm. Sci.* **160**, 105772 (2021). 33621612 10.1016/j.ejps.2021.105772

[26] Mathias, E. V., Aponte, J., Kornfield, J. A. & Ba, Y. Properties of small molecular drug loading and diffusion in a fluorinated PEG hydrogel studied by 1 H molecular diffusion NMR and 19 F spin diffusion NMR. *Colloid Polym. Sci.* **288**, 1655–1663 (2010). 21170115 10.1007/s00396-010-2304-9 PMC2982959

[27] Bálint, Š. et al. Diffusion and cellular uptake of drugs in live cells studied with surface-enhanced Raman scattering probes. *J. Biomed. Opt.* **15**, 027005–027005 (2010). 20459279 10.1117/1.3369844

[28] Abrahams, M. S., Panzer, O., Atchabahian, A., Horn, J. L. & Brown, A. R. Case report: limitation of local anesthetic spread during ultrasound-guided interscalene block. Description of an anatomic variant with clinical correlation. *Reg. Anesth. Pain Med.* **33**, 357–359 (2008). 18675748 10.1016/j.rapm.2008.01.015

[29] Tsutsumi, A., Umehara, K., Ono, H. & Kawakami, N. Types of psychosocial job demands and adverse events due to dental mismanagement: a cross sectional study. *BMC Oral Health*. **7**, 1–6 (2007). 17408488 10.1186/1472-6831-7-3 PMC1854897

[30] Šimek, J., Šmejkal, K., Jakl, M. & Trlica, J. Systemic toxic effects of Mesocain^®^ in routine surgical practice upon iatrogenic overdose requiring cardiopulmonary resuscitation-case report. *Rozhledy V Chirurgii: Mesicnik Ceskoslovenske Chirurgicke Spolecnosti*. **102**, 257–260 (2024). 10.33699/PIS.2023.102.6.257-260 38286655

[31] Wong, S. et al. Image analysis comparison of nerve staining with food dye, methylene blue or tissue marker. *Veterinary Anaesth. Analg.* **51**, 35–43 (2024). 10.1016/j.vaa.2023.09.073 38016893

[32] Zhao, J. et al. Tissue-Selective separation mechanism of Multi-type soft tissues under waterjet impact for Low-Trauma cutting surgery. *J. Ann. Biomed. Eng.* **53**, 2612–2625 (2025). 10.1007/s10439-025-03801-3 40684057

[33] Derakhshan, R., Ahmadian, M., Chizari, M. & Samimiardestani, H. Trimming of sheep spinal cord by waterjet; an experimental study. *Heliyon* **9**, 2563 (2023). 10.1016/j.heliyon.2023.e17872 PMC10366390 37496918

[34] Cui, D. et al. Experimental study on the effect of water jet cutting parameters on maize stalks. *Agriculture* **13**, 880 (2023).

[35] Popan, I. A., Contiu, G. & Campbell, I. In *MATEC Web of Conferences.* 01009 (EDP Sciences, 2025).

[36] Peneva, M., Gjorgjeska, A., Ginoski, V., Breshkovska, H. & Tolevska, R. D. Electrosurgical microneedle versus scalpel skin incisions in the facial region. *Sanamed* **13**, 269–273 (2018). 10.2478/prilozi-2018-0048 30864361

[37] Ravi, R. R. & Srinivasu, D. Jet pressure influence on micro-abrasive waterjet trepanned hole in CFRP composite material. *Manuf. Technol. Today*. **22**, 24–29 (2023).

[38] Zeng, D., Tang, Z., Wang, W., Wang, Z. & Li, J. Experimental investigation of the optimal driving pressure for a larger-volume controllable jet injection system. *Med. Eng. Phys.* **119**, 104033 (2023). 37634910 10.1016/j.medengphy.2023.104033

[39] Zhongwei, H., Wangyuan, L. & Xipeng, X. Experimental study on the cutting characteristics of three typical biological soft tissues. *J. Mech. Eng.* **52**, 186–192 (2016).

[40] Liu, Z. et al. Recent advances in soft biological tissue manipulating technologies. *Chin. J. Mech. Eng.* **35**, 89 (2022).

[41] Wong, S. L. et al. Sentinel lymph node biopsy and management of regional lymph nodes in melanoma: American society of clinical oncology and society of surgical oncology clinical practice guideline update. *J. Clin. Oncol.* **36**, 399–413 (2018). 29232171 10.1200/JCO.2017.75.7724

[42] Muldrew, K. et al. Cryobiology of articular cartilage: ice morphology and recovery of chondrocytes. *Cryobiology* **40**, 102–109 (2000). 10788309 10.1006/cryo.2000.2236

[43] Prescher, H. et al. Scalpel edge roughness affects post-transection peripheral nerve regeneration. *Surg. Open* **4**, 1–6 (2021). 10.1016/j.sopen.2020.11.002 PMC7838729 33537665

[44] Casutt, M. M., Scheeder, M. R., Escher, F., Dufey, P. A. & Kreuzer, M. Relating texture properties and composition of bovine fat tissue. *Lipid/Fett* **101**, 283–290 (1999).

[45] Sidorov, D. et al. Surgical and pathomorphological results of total mesorectumectomy by using waterjet dissection technique in patients with rectal cancer. *Surg. Oncol.* **6**, 17–22 (2016).

